# The benefits of pair bond tenure in the cooperatively breeding pied babbler (*Turdoides bicolor*)

**DOI:** 10.1002/ece3.4243

**Published:** 2018-06-11

**Authors:** Elizabeth M. Wiley, Amanda R. Ridley

**Affiliations:** ^1^ Centre for Evolutionary Biology School of Biological Sciences University of Western Australia Crawley WA Australia; ^2^ Department of Zoology Percy FitzPatrick Institute of African Ornithology University of Cape Town Rondebosch South Africa

**Keywords:** cooperative breeding, pair bond, pair tenure, pied babblers, reproductive success

## Abstract

The benefits of stable pair bonds (that persist between breeding attempts) have been well described, but are relatively less well known in cooperatively breeding species. If pair bonds are beneficial, then it is possible that the bond between the behaviorally and socially dominant pair may influence factors such as reproductive success and group stability in cooperative species. Here, we used long‐term data to investigate the relationships between pair bond tenure, reproductive success, and group stability in the cooperatively breeding pied babbler (*Turdoides bicolor*). Pair bond tenure positively influenced both the number of offspring recruited *annually* per pair and total reproductive success (over entire pair bond duration), indicating that pair bond tenure has an important influence on reproductive success. The likelihood of immigration into the group was lower for groups containing a bonded pair with long tenure, indicating that the duration of pair bonds may impact group stability. These findings suggest that pair tenure, a hitherto relatively unexplored factor in cooperative species, may have an important influence on group dynamics.

## 1 INTRODUCTION

Long‐term sequential monogamy, where a pair stay together for consecutive reproductive attempts, has been well described in biparental species (reviewed in Wittenberger & Tilson, 1980; Dillard & Westneat, 2016). Pairs that form and maintain pair bonds, rather than repeatedly divorcing and remating, may reap fitness benefits from such long‐term bonds (Forslund & Larsson, 1991). Increased mate familiarity over time through pair bonding may also facilitate better coordination in reproductive activities, territory acquisition and defence, and antipredator behaviors, for example, barnacle geese, *Branta leucopsis* (Black, 2001); blue‐footed boobies, *Sula nebouxii* (Sánchez‐Macouzet, Rodríguez, & Drummond, 2014).

Monogamous pair bonds can also be present in cooperative species, where the breeding pair in a cooperative group stay together over an extended time period (e.g., cichlids, *Neolamprologus pulcher* (Bergmüller, Heg, & Taborsky, 2005); red wolves, *Canis rufus* (Sparkman, Adams, Steury, Waits, & Murray, 2011)). For many cooperative breeders, reproductive success is an important predictor of annual group persistence, due to the benefits accrued through group augmentation (Keynan & Ridley, 2016; Kokko, Johnstone, & Clutton‐Brock, 2001; Wiley, 2017). Groups can also increase in size through the recruitment of unrelated individuals; however, nonkin group compositions can be more likely to lead to within‐group conflict due to reproductive competition (Goldstein, Woolfenden, & Hailman, 1998; Leimar & Hammerstein, 2010; Nelson‐Flower & Ridley, 2016; Ridley, 2016). In cooperative species, the bond between sequentially monogamous pairs may be important to the stability of the group, via direct influences on reproductive success, immigration, and within‐group conflict. Thus far, this possibility has rarely been investigated.

Pied babbler pairs form monogamous long‐term pair bonds within groups, with very low extrapair parentage: 92.3% of offspring are progeny of the dominant pair (Nelson‐Flower, Flower, & Ridley, 2018). Pied babblers are a long‐lived passerine, with some individuals reaching more than 10 years of age in the wild (Ridley, 2016); thus, pair bonds can persist for many years. Pied babblers live in stable groups consisting of a *single* breeding pair (Nelson‐Flower et al., 2011), and sexually mature (over 1 year old posthatching) subordinate helpers, with an average group size (±standard error) of 4.29 ± 0.22 adults (range: 2–13 adults, Wiley, 2017).

Here, we aim to test whether the prevailing trend seen in biparental species, where longer pair bond tenure results in enhanced reproductive success (Black, 1996; Griggio & Hoi, 2011; Sánchez‐Macouzet et al., 2014; Wittenberger & Tilson, 1980), persists in cooperative species. We also aim to test the idea that longer term pair bonds confer *group stability* in cooperative species, analogous to the *territory stability* observed in biparental or pair bonded species with long‐term partner fidelity (Hall & Magrath, 2007; Nowicki et al., 2018). In some social species, immigration events are known to negatively impact the stability of groups, with consequences such as the eviction or infanticide of group members (Packer, Scheel, & Pusey, 1990; Silk, 2007). We therefore use the relationship between pair bond tenure and group immigration events as a proxy for group stability. We investigate the effect of pair bond tenure on reproductive success and group stability in the pied babbler by quantifying reproductive success, pair persistence (likelihood of remaining as a pair to the next breeding season), and group immigration events over the duration of the pair bond.

## 2 METHODS

### 2.1 Study site

We investigated monogamous pair bonds in pied babbler groups at the Pied Babbler Research Project, based in the 33 km^2^ Kuruman River Reserve, southern Kalahari, South Africa (26°58′S, 21°49′E). The study site has a subtropical climate and is primarily semi‐arid grassland and acacia savanna (see Ridley & Thompson, 2011 for description of habitat types).

### 2.2 Study species

**Figure 1:**
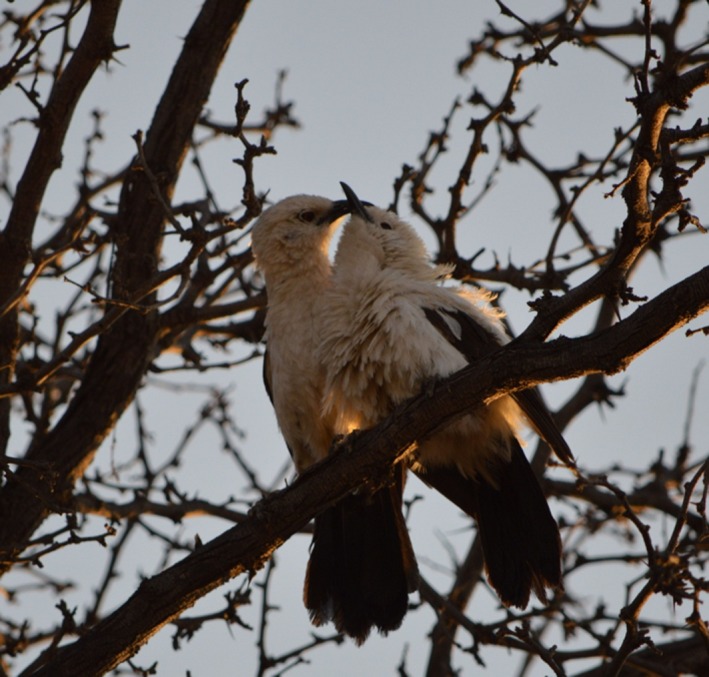
A pied babbler pair allopreening before going to roost. Tactile affiliative interactions between individuals may serve to strengthen social bonds. Observations of behavior can be recorded at a distance of 1–2 m from these habituated birds. (Photo by EMWiley)

The pied babbler is a cooperatively breeding, territorial, medium‐sized (75–95 g) passerine, in which all adult group members contribute to the provisioning of nestlings and fledglings (Ridley & Raihani, 2007). Pied babblers can raise up to three broods per season. Breeding normally occurs in summer months but can occur year‐round if ecological conditions permit (Ridley, 2016). Since 2003, a study population of uniquely ringed individuals has been habituated, monitored and maintained at the study site, typically comprising 18 habituated groups of pied babblers each year. Groups are visited at least once per week leading up to and during the peak breeding season (September–March), to check group composition and record life history events such as breeding, immigration, and dispersal. For a small food reward, individuals will hop onto a small top‐pan scale to be weighed. In this way, body condition can be monitored throughout each individual's lifetime noninvasively, thus avoiding any need for recapture. Individuals typically must be part of the dominant breeding pair (one such pair per group) in order to breed (Nelson‐Flower et al., 2011). Although subordinate reproduction does occur, it is very rare (Nelson‐Flower, Flower, et al., 2018). In each group, the dominant pair enforce their dominance through agonistic displays and physical attacks on subordinates (Raihani, 2008). The dominant pair is also readily identifiable through regular duetting and affiliative behavior (Golabek, 2010; Wiley, 2017) and as the primary individuals engaging in nest‐building and breeding activity (Nelson‐Flower et al., 2013). Thus assignation of the breeding pair in each group is readily determined and unambiguous (Figure 1). Age of acquisition of dominance varies between males and females, with females typically acquiring dominance at 882 days posthatching (range 204–2,761), while males typically acquire dominance at 1,085 days posthatching (range 319–2,679). Inbreeding avoidance in this species results in the regular dispersal of individuals from their natal group to access breeding opportunities (Nelson‐Flower, Hockey, O'Ryan, & Ridley, 2012). Genetic research has revealed that individuals will gain the dominant breeding position on their natal territory only if the opposite‐sex dominant individual is not a close relative (Nelson‐Flower, Wiley, Flower, & Ridley, 2018).

Long‐term monitoring of cooperative behavior and lifetime reproductive success in the population precluded experimental manipulation of breeding pairs. However, by controlling for body mass and age we were able to account, at least in part, for the potential confounds of variation in individual quality acting on pair tenure and reproductive success.

### 2.3 Data collection

For every pair monitored from 2003 to 2015, individual data on the *sex*,* age* (in days since hatching) of each member of the pair, *body mass* (an average of all morning mass measurements per individual per year) and *previous breeding experience* as an individual (total breeding attempts. An attempt being defined as when eggs were laid and incubated, per individual regardless of current pairing) were extracted from the long‐term database.

For each pair, data on the *pair length* (total consecutive days since pair bonded, cumulative across years), *previous reproductive success* as a pair (total offspring recruited to one year of age, cumulative across years extant as a pair), *group size* (adults present at the start of each year), *likelihood of pair persistence* (whether a pair was still together the next year (yes/no)), *number of immigrants* (number of new adult individuals immigrating into the group per year: Individuals that spent more than 30 consecutive days in a group were considered to have successfully immigrated) and *annual chick recruitment* (number of offspring produced by the pair that survived to at least 1 year for each year the pair were together) were extracted. Each “year” started on 1 September (at the beginning of the breeding season) and ended on 31 August the next year.

There were a total of 64 pairs and 86 individuals available for analysis, of which 47 were female and 39 male. There was a difference in the number of males and females available for analysis because of differential mortality between the sexes. Where possible, we used known age (from hatching records). However, there were four (female) individuals that did not have an exact age. For these four, we assigned to them the average age at which a female attained a dominant position in a group (882 days, calculated from the other 43 females that acquired dominance rank) as their age on the date they became dominant, in the first year that they were part of a known breeding pair in the study population.

Pair lifetime reproductive success was estimated as the total number of offspring (that survived to 1 year of age) recruited over the duration of the pair tenure. We also calculated the total number of offspring (that survived to 1 year) produced from the total number of hatched broods per pair over the duration of the pair tenure. Calculating this way measured reproductive output per (viable) nesting attempt and thus avoided having time as a confounding factor. These analyses comprised a subset of pairs from the database (*n* = 57) because this variable could only be calculated for pair bonds that were no longer extant.

### 2.4 Statistical analysis

#### 2.4.1 Chick recruitment

To determine which variables influenced the number of chicks each pair recruited per year, generalized linear mixed models (hereafter GLMMs) with Poisson distributions were employed for males and females separately. Previous reproductive success (as a pair; some individuals were present as members of different pairs at different times, thus offspring produced *per pairing* was analyzed separately), previous breeding experience, breeder age, group size, body mass and pair tenure were included as predictor terms. Year and individual identity nested within pair identity were included as random effects in all models.

#### 2.4.2 Pair persistence

To determine which variables influenced pair persistence likelihood per year, GLMMs with binomial distributions (where 0 = pair bond ended, 1 = pair bond remained extant) were used with breeder age, group size, body mass, chick recruitment, previous reproductive success and pair tenure as predictor terms. Year and individual identity nested within pair identity were included as random effects in all models.

#### 2.4.3 Reproductive success over entire duration of the pair bond

To determine what was influencing reproductive success (total offspring recruited to 1 year of age over entire pair tenure), GLMMs with Poisson distributions were employed, with average group size (average number of adults in the group for the duration of the pair tenure), and pair tenure (total days) as predictor terms. Group identity and year were included as random effects.

To investigate which parameters were affecting reproductive success (per hatched brood), we analyzed data in LMMs, with average group size (average number of adults in the group for the duration of the pair tenure), and pair tenure (total days) as predictor terms. Group identity and year were included as random effects.

#### 2.4.4 Group stability

We investigated factors influencing immigration events into a group as a measure of group stability, using GLMMs with Poisson distributions, with pair tenure (total days), chick recruitment (total number of offspring recruited each year) and group size (adult group size in each year of pair tenure) as predictor terms. Pair identity nested within group identity and year was included as random effects.

Data from paired males and females were analyzed separately where individual‐level data were used as predictor terms in models, to avoid a lack of independence in the predictor variables of annual chick recruitment or pair persistence values. Correlated terms were not used together in the same models (see Supporting Information Table S7).

Model selection using the Akaike's information criterion corrected for small sample size (AICc) was employed to determine the model/s that best explained the patterns of variation in the data. Using AICc (with maximum likelihood estimation) a series of models were tested, with each model representing a biological hypothesis. Lower AICc values represented more parsimonious models, as per Johnson and Omland (2004). The best‐supported models were selected and where there were several models within 2AICc, the model with the fewest explanatory terms, that is, the simplest, was selected (Burnham & Anderson, 2003). All data were analyzed in the program “R” v 3.3.2 (2017), using the package “lme4” (Bates, Maechler, Bolker, & Walker, 2015). All continuous explanatory variables were scaled following Grueber, Nakagawa, Laws, & Jamieson, 2011 to allow model comparison, using the function “scale” in the program “R” (2017).

## 3 RESULTS

### 3.1 Pair bond tenure and reproductive success

**Figure 2:**
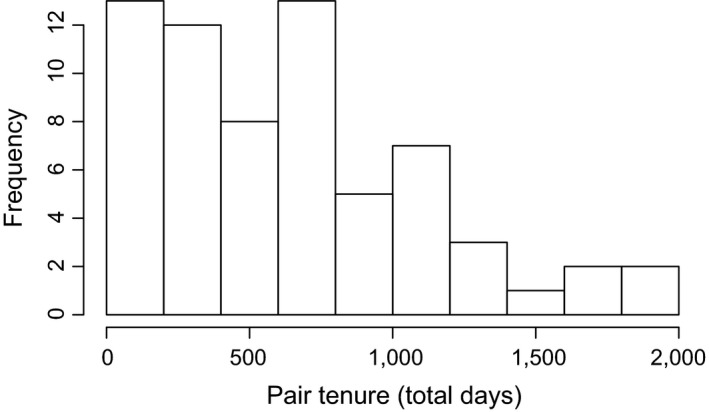
The frequency and distribution of known pair tenures in the pied babbler population, from 2003 to 2015

In pied babblers, completed pair bonds (where the pair bond had ended and total pair tenure length was known) averaged 609 days, and ranged widely, from 19 to 1,940 days (Figure 2). On average, males formed pair bonds with 1.5 females over their lifetime, while females on average formed pair bonds with an average of 1.3 males.

**Figure 3:**
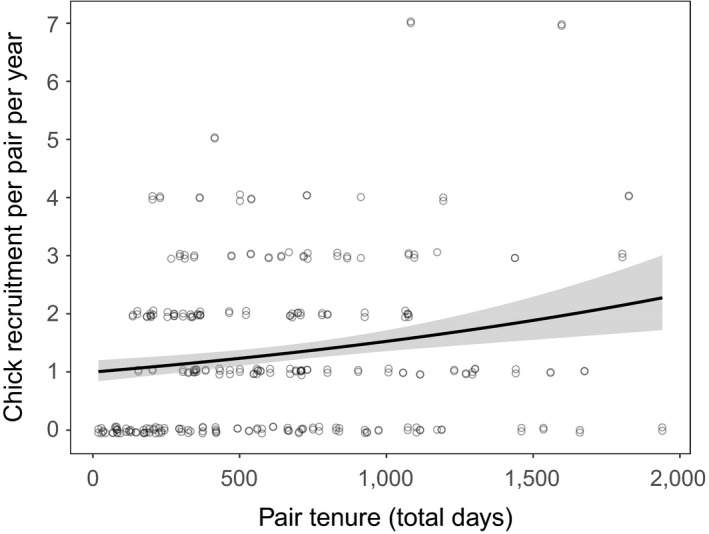
The relationship between chick recruitment (per year) and pair tenure. The fitted regression line is shown with shaded 95% confidence intervals. Data points are integers and have been jittered for better visibility

Pairs with longer tenure had significantly higher chick recruitment per year (Figure 3, Table 1 and for full model lists see Supporting Information Tables S1 and S2).

**Table 1:** The top model set for the GLMM analysis of the terms influencing the number of chicks successfully recruited to 1 year of age per year, for females and males separately

Model	AICc	ΔAICc	ωί
**Females**			
Pair length	453.96	0.57	0.42
Pair length + body mass	453.39	0	0.55
Null	462.48	9.09	0.01
**Parameter estimates**	**Estimate**	***SE***	***Z***
Intercept	0.18	0.18	0.97
Pair length	0.26	0.07	3.62
**Males**			
Pair length	448.09	0.04	0.48
Pair length + body mass	448.06	0	0.49
Null			
**Parameter estimates**	**Estimate**	***SE***	***Z***
Intercept	0.19	0.18	1.05
Pair length	0.27	0.07	3.74

### 3.2 Pair persistence

**Figure 4:**
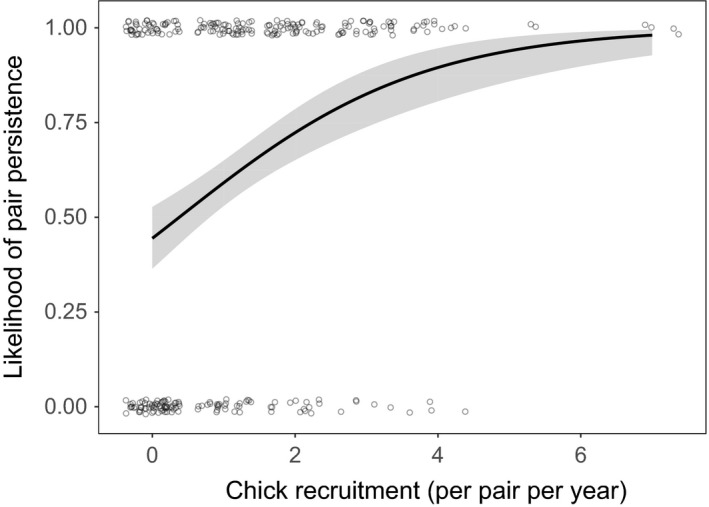
The relationship between the number of chicks (raised to 1 year of age posthatch) and the likelihood a pair persisted to the next year. A value of “1” on the *y*‐axis denotes persistence. The fitted regression line is shown with shaded 95% confidence intervals. Data points are integers and have been jittered for better visibility

Pairs that successfully raised chicks that survived to 1 year of age were more likely to still be a pair in the following year (Figure 4, Table 2 and for full model lists see Supporting Information Tables S3 and S4).

**Table 2:** The top model set for the GLMM analysis of the terms influencing the likelihood of pair persistence to the following year, for females and males separately

Model	AICc	ΔAICc	ωί
**Females**			
Chick recruitment	190.28	0	0.99
Null	204.21	13.92	0
**Parameter estimates**	**Estimate**	***SE***	***Z***
Intercept	0.55	0.19	2.83
Chick recruitment	0.83	0.23	3.57
**Males**			
Chick recruitment	188.07	0	1
Null	200.21	12.14	0
**Parameter estimates**	**Estimate**	***SE***	***Z***
Intercept	0.55	0.19	2.88
Chick recruitment	0.81	0.23	3.51

### 3.3 Reproductive success (total pair duration)

**Figure 5:**
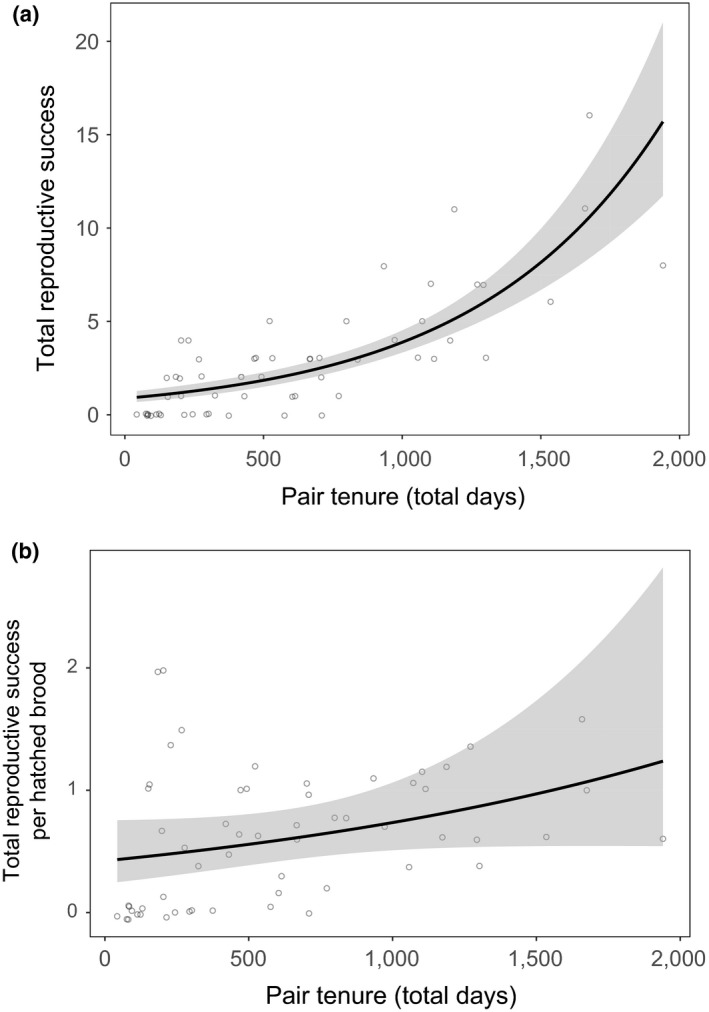
(a) The relationship between reproductive success (total number of chicks reaching adulthood that a pair raise together) and complete pair tenure (total length in days). The fitted regression line is shown with shaded 95% confidence intervals. Data points are integers and have been jittered for better visibility. (b) The relationship between total reproductive success per hatched brood (over entire pair duration) and complete pair tenure (total length in days). The fitted regression line is shown with shaded 95% confidence intervals. Data points are integers and have been jittered for better visibility

Patterns of pair lifetime reproductive success were best explained by pair tenure for both (a) total reproductive success and (b) reproductive success per hatched brood (Figures 5a and 5b, Table 3 and for a full list of models tested, see Supporting Information Tables S5 and S6).

**Table 3:** The top model set for the GLMM analysis of the terms influencing within‐pair lifetime reproductive success and within‐pair lifetime reproductive success as a proportion of hatched broods

Model	AICc	ΔAICc	ωί
**Lifetime reproductive success**			
Pair length	215.49	0	1
Null	275.96	60.47	0
**Parameter estimates**	**Estimate**	***SE***	***Z***
Intercept	0.74	0.12	6.18
Pair length	0.71	0.07	9.001
**Lifetime reproductive success per hatched brood**			
Pair length	35.77	0	1
Null	44.14	8.37	0
**Parameter estimates**	**Estimate**	***SE***	***X*** ^**2**^
Intercept	0.42	0.04	
Pair length	0.13	0.04	10.64

### 3.4 Group stability

**Figure 6:**
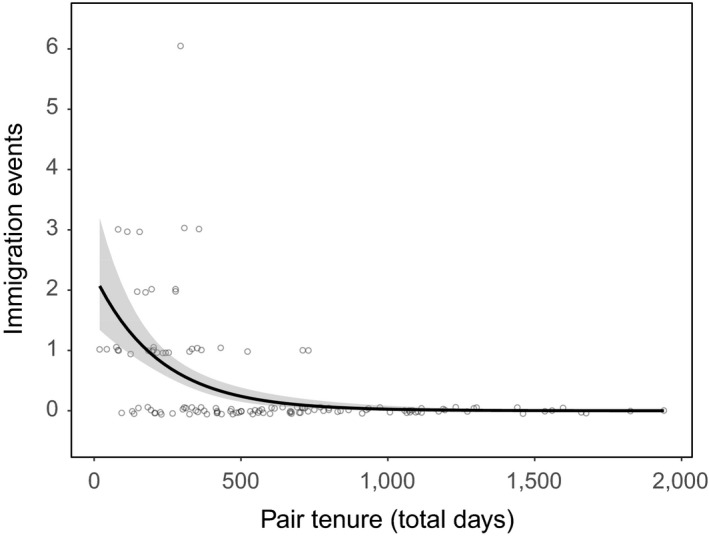
The relationship between immigration events and complete pair tenure (total length in days). The fitted regression line is shown with shaded 95% confidence intervals. Data points are integers and have been jittered for better visibility

Immigration events were less likely to occur at groups where the bonded pair had longer pair tenure (Figure 6 and Table 4). This effect was so strong, that groups with pairs that were bonded for more than 2 years (730 days) experienced less than 2% of all the immigration events observed in the population (*n* = 52 immigration events), despite the fact that pairs bonded for this long comprised more than 30% of our dataset.

**Table 4:** The top model set for the GLMM analysis of the terms influencing immigration to the group for each year of each pair bond tenure. Analysis was conducted on the total number of immigration events in each year a pair were extant. The best‐supported model is bolded

Model	*df*	AICc	ΔAICc	ωί	logLik
Pair length	**5**	**178.98**	**0**	**1**	**−84.24**
Chick recruitment	5	223.44	44.46	0	−106.47
Null	4	224.38	45.40	0	−108.02
Group size	5	224.53	45.55	0	−107.01

Parameter estimates	Estimate	*SE*	*z*
Intercept	−2.27	0.42	−5.45
Pair length	−2.11	0.41	−5.09

*Note*

*N* = 126. Random effects: Year: 13 (*SD* 0), Group ID: 18 (*SD* 0), Group ID/Pair ID: 57 (*SD* 0.69).

## 4 DISCUSSION

The benefits of pair bonding have been widely studied and the general consensus has been that the function of monogamous pair bonds is to increase reproductive output (Bradley, Wooller, & Skira, 1995; Evans & Poole, 1983; Fowler, 1995). Under the recently proposed dual benefits framework (Shen, Emlen, Koenig, & Rubenstein, 2017), cooperation is associated with “collective action benefits” (which include increased per capita productivity), but the potential benefits of pair bonding for productivity in cooperatively breeding species has received relatively little attention. In this study, we were able to confirm several substantial benefits of pair bonds in a cooperatively breeding species.

First, reproductive success was higher for those individuals with a longer pair bond. Duration of pair tenure also had a positive influence on within‐pair *lifetime* reproductive success. This supports findings from biparental species (Black, 1996; Wittenberger & Tilson, 1980), but provides one of the first instances of this benefit in a cooperative species. Additionally, an increase in reproductive success per year may imply that long‐term pair bonds in pied babblers accrue benefits through mate familiarity. Although further testing is required, this could corroborate similar results in species with biparental care such as Steller's Jays, *Cyanocitta stelleri* (Gabriel & Black, 2013) and Pinyon Jays, *Gymnorhinus cyanocephalus* (Marzluff & Balda, 1988) in which longer lasting pairs had higher reproductive success (independent of individual age).

Second, annual reproductive success increased the likelihood of a pair still being bonded in the next breeding season. This result suggests that reproductive success is not only a benefit of pair tenure, but (barring the death of one partner) could also be a key determinant in whether a pair continues to remain bonded. This result supports findings from a recent meta‐analysis of monogamous bird species, where divorce was suggested to be an adaptive strategy to improve poor breeding success (Culina, Radersma, & Sheldon, 2015).

Our results revealed a significant impact of pair bond tenure on immigration events, suggesting a significant impact of pair bond tenure on group stability—a finding that has hitherto not been reported for cooperative species. In pied babblers, there is a greater likelihood of within‐group conflict in groups comprised of nonkin, where reproductive competition decreases group productivity (Nelson‐Flower et al., 2013). Thus, where a long‐term pair bond precluded the immigration of unrelated individuals, within‐group stability was high, possibly due to lower intragroup reproductive competition. Our analysis therefore reveals that there are selective benefits associated with pair bond tenure in a cooperatively breeding species.

Longer pair bonds resulted in both higher lifetime, but also higher annual reproductive success, and this in turn positively influenced pair persistence to the following breeding season. Pairs with longer tenure also gained fewer adult immigrants into their group, thus possibly minimizing the potential for within‐group conflict due to reproductive success. Pair bonds may therefore benefit the whole group: Higher reproductive success can confer direct (production of individual's own offspring) or indirect (individual is related to offspring produced) fitness benefits to group members (Clutton‐Brock, 2002). Pair bonds are a relatively unexplored aspect of the complex social dynamics of cooperative breeders, and we have found, using an extensive long‐term dataset that encompasses lifetime pairing for many individuals, that pair tenure may have an important influence on both within‐pair and within‐group dynamics of a cooperatively breeding species.

## CONFLICT OF INTEREST

The authors declare we have no competing interests.

## AUTHOR CONTRIBUTIONS

EMW and ARR conceived the study idea and contributed to data collection. EMW carried out data analysis and wrote the manuscript. ARR habituated the study population, maintained long‐term data collection at the field site, and critically contributed to manuscript drafts.

## ETHICAL NOTE

Our research was approved by the Animal Ethics Committee, University of Western Australia (RA/3/100/1263) and the Science Faculty Animal Ethics Committee, University of Cape Town (Ethics number R2012/2006/V15/AR).

## DATA ACCESSIBILITY

The datasets supporting this article have been deposited in Dryad https://doi.org/10.5061/dryad.r3mk2ng
