# Supplementary material for: The benefits of pair bond tenure in the cooperatively breeding pied babbler (Turdoides bicolor)
Source: Ecol Evol. 2018 Jun 11;8(14):7178–85. doi: 10.1002/ece3.4243 (PMC6065330; doi:10.1002/ece3.4243)
Supplement: Supplementary file 1 [file ECE3-8-7178-s001.docx]

Benefits of pair bond supplementary material (EMW, ARR)


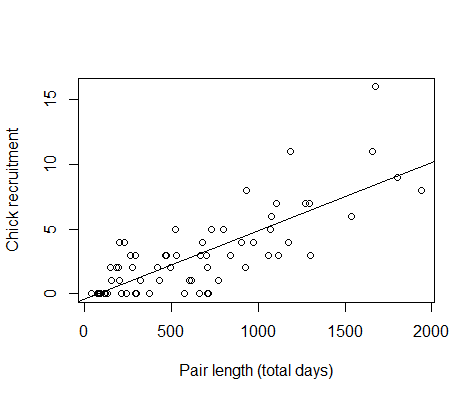


Figure S1. The relationship between chick recruitment and pair tenure, the significant linear model fit line is shown. (Pearson’s r=0.783, n=64, p=<0.0001).

*Chick recruitment*

Table S1. Model terms tested for influencing the number of chicks successfully recruited per year for females in a bonded pair. Analysis was conducted on the number of chicks recruited yearly by 47 females in 64 pairs in 22 groups over 13 years. The model in bold was selected as the simplest of the best supported models. A full model is not presented as correlated terms were not included in the same submodels.

| Model | df | AICc | ΔAICc | ωί | logLik |
| --- | --- | --- | --- | --- | --- |
| **Pair tenure** | **5** | **453.96** | **0.57** | **0.42** | **-221.77** |
| Pair tenure + body mass | 6 | 453.39 | 0 | 0.55 | -220.39 |
| Body mass | 5 | 461.90 | 8.51 | 0.01 | -225.74 |
| Body mass + previous reproductive success | 6 | 462.44 | 9.05 | 0.01 | -224.92 |
| Null | 4 | 462.48 | 9.09 | 0.01 | -227.10 |
| Previous reproductive success | 5 | 463.25 | 9.87 | 0 | -226.41 |
| Body mass + breeding experience | 6 | 463.87 | 10.48 | 0 | -225.64 |
| Age | 5 | 464.38 | 10.99 | 0 | -226.97 |
| Breeding experience | 5 | 464.57 | 11.18 | 0 | -227.07 |
|  |  |  |  |  |  |
| **Parameter estimates** |  | **estimate** | **se** | **z** |  |
| Intercept |  | 0.18 | 0.18 | 0.97 |  |
| Pair tenure |  | 0.26 | 0.07 | 3.62 |  |

N=147. Random effects: Year: 13 (SD 0.47), Pair ID/ Individual ID: 64 (SD 0.05)

Table S2. Model terms tested for influencing the number of chicks successfully recruited per year for males in a bonded pair. Analysis was conducted on the number of chicks recruited yearly by 39 males in 63 pairs in 21 groups over 13 years. The model in bold was selected as the simplest of the best supported models.

| Model | df | AICc | ΔAICc | ωί | logLik |
| --- | --- | --- | --- | --- | --- |
| **Pair tenure** | **5** | **448.09** | **0.04** | **0.48** | **-217.72** |
| Pair tenure + body mass | 6 | 448.06 | 0 | 0.49 | -218.83 |
| Body mass | 5 | 456.30 | 8.24 | 0.01 | -222.93 |
| Null | 4 | 457.48 | 9.42 | 0 | -224.59 |
| Body mass + breeding experience | 6 | 457.68 | 9.62 | 0 | -222.72 |
| Body mass + previous reproductive success | 6 | 458.04 | 9.99 | 0 | -223.99 |
| Previous reproductive success | 5 | 458.41 | 10.35 | 0 |  |
| Breeding experience | 5 | 459.51 | 11.45 | 0 |  |
| Age | 5 | 459.62 | 11.56 | 0 |  |
|  |  |  |  |  |  |
| **Parameter estimates** |  | **estimate** | **se** | **z** |  |
| Intercept |  | 0.19 | 0.18 | 1.05 |  |
| Pair tenure |  | 0.27 | 0.07 | 3.74 |  |

N=145. Random effects: Year: 13 (SD 0.57), Pair ID/ Individual ID: 63 (SD 0.07)

*Pair persistence*

Table S3. Model terms tested for influencing the likelihood of pair persistence to the next year for females. Analysis was conducted on the annual reproductive success of 47 females in 64 pairs in 22 groups over 13 years. The best supported model is bolded.

| Model | df | AICc | ΔAICc | ωί | logLik |
| --- | --- | --- | --- | --- | --- |
| **Chick recruitment** | **5** | **190.28** | **0** | **0.99** | **-89.93** |
| Previous reproductive success | 5 | 202.95 | 12.67 | 0 | -96.26 |
| Previous reproductive success + body mass | 6 | 203.85 | 13.57 | 0 | -95.63 |
| Null | 4 | 204.21 | 13.92 | 0 | -97.96 |
| Body mass | 5 | 204.96 | 14.68 | 0 | -97.27 |
| Age | 5 | 205.34 | 15.06 | 0 | -97.46 |
| Group size | 5 | 206.06 | 15.78 | 0 | -97.82 |
| Pair tenure | 5 | 206.34 | 16.06 | 0 | -97.96 |
| Body mass + pair tenure | 6 | 207.13 | 16.85 | 0 | -97.27 |
|  |  |  |  |  |  |
| **Parameter estimates** |  | **estimate** | **se** | **z** |  |
| Intercept |  | 0.55 | 0.19 | 2.83 |  |
| Chick recruitment |  | 0.83 | 0.23 | 3.57 |  |

N=147. Random effects: Year: 13 (SD 0.19), Pair ID/ Individual ID: 64 (SD 0)

Table S4. Model terms tested for influencing the likelihood of pair persistence to the next year for males. Analysis was conducted on the annual reproductive success of 39 males in 63 pairs in 21 groups, over 13 years. The best supported model is bolded.

| Model | df | AICc | ΔAICc | ωί | logLik |
| --- | --- | --- | --- | --- | --- |
| **Chick recruitment** | **5** | **188.07** | **0** | **1** | **-88.82** |
| Previous reproductive success | 5 | 200.21 | 12.14 | 0 | -94.89 |
| Null | 4 | 201.63 | 13.57 | 0 | -96.67 |
| Previous reproductive success + body mass | 6 | 202.39 | 14.32 | 0 | -94.89 |
| Age | 5 | 202.50 | 14.44 | 0 | -96.03 |
| Body mass | 5 | 203.76 | 15.69 | 0 | -96.66 |
| Pair tenure | 5 | 203.77 | 15.70 | 0 | -96.67 |
| Group size | 5 | 203.77 | 15.71 | 0 | -96.67 |
| Body mass + pair tenure | 6 | 205.93 | 17.86 | 0 | -96.66 |
|  |  |  |  |  |  |
| **Parameter estimates** |  | **estimate** | **se** | **z** |  |
| Intercept |  | 0.55 | 0.19 | 2.88 |  |
| Chick recruitment |  | 0.81 | 0.23 | 3.51 |  |

N=145. Random effects: Year: 13 (SD 0.14), Pair ID/ Individual ID: 63 (SD 0)

*Reproductive success*

Table S5. Model terms tested for influencing pair lifetime total reproductive success. Analysis conducted on the total number of offspring successfully recruited by each of 57 pairs over a 13-year period, where total pair tenure was known and pairs were no longer extant. The best supported model is bolded.

| Model | df | AICc | ΔAICc | ωί | logLik |
| --- | --- | --- | --- | --- | --- |
| **Pair tenure** | **4** | **215.49** | **0** | **1** | **-103.36** |
| Average adult group size | 4 | 267.80 | 52.31 | 0 | -129.52 |
| Null | 3 | 275.96 | 60.47 | 0 | -134.75 |
|  |  |  |  |  |  |
| **Parameter estimates** |  | **estimate** | **se** | **z** |  |
| Intercept |  | 0.74 | 0.12 | 6.18 |  |
| Pair tenure |  | 0.71 | 0.07 | 9.001 |  |

N=57. Random effects: Year: 13 (SD 0.04), Group ID: 18 (SD 0.25)

Table S6. Model terms tested for influencing pair lifetime reproductive success as a proportion of total hatched broods. Analysis conducted on the total number of offspring successfully recruited by each of 57 pairs over a 13-year period, where total pair tenure was known and pairs were no longer extant. The best supported model is bolded.

| Model | df | AICc | ΔAICc | ωί | logLik |
| --- | --- | --- | --- | --- | --- |
| **Pair length** | **5** | **35.77** | **0** | **1** | **-12.88** |
| Null | 4 | 42.79 | 7.02 | 0 | -17.39 |
| Average group size | 5 | 44.14 | 8.37 | 0 | -17.07 |
|  |  |  |  |  |  |
| **Parameter estimates** |  | **estimate** | **se** | **X^2^** |  |
| Intercept |  | 0.42 | 0.04 |  |  |
| Pair length |  | 0.13 | 0.04 | 10.64 |  |

N=57. Random effects: Year: 13 (SD 0), Group ID: 18 (SD 0.03)

Table S7. Correlated terms (calculated using Spearman’s rank test) which were not used together in models.

| Correlated terms | *rho* | n | p |
| --- | --- | --- | --- |
| Previous RS & pair length | 0.800 | 290 | <0.001 |
| Previous RS & Breeding exp | 0.610 | 290 | <0.001 |
| Previous RS & group size | 0.317 | 290 | <0.001 |
| Previous RS & age | 0.427 | 290 | <0.001 |
| Breeding exp & pair length | 0.542 | 290 | <0.001 |
| Pair length & age | 0.468 | 290 | <0.001 |
| Breeding exp & age | 0.551 | 290 | <0.001 |
| Group size and pair length | 0.416 | 290 | 0.001 |
